# Antiseptic Techniques in Breast Implant Surgery: Insights From Plastic Surgeons in Saudi Arabia

**DOI:** 10.1093/asjof/ojad077

**Published:** 2023-08-25

**Authors:** Hatan Mortada, Faisal Falah Almutairi, Saad Alrobaiea, Ayman M Helmi, Abdullah E Kattan, Adnan G Gelidan, Khalid Arab

## Abstract

**Background:**

Breast implant surgery is a popular procedure worldwide, and the same holds true for Saudi Arabia. Ensuring a sterile surgical environment is crucial to avert postoperative infections. This study explores the various antiseptic techniques adopted by Saudi plastic surgeons during breast implant procedures.

**Objectives:**

This study aims to assess Saudi plastic surgeons’ adherence to antiseptic measures in breast implant surgery, and determine what types of antiseptic measures are most commonly used among Saudi plastic surgeons.

**Methods:**

The authors conducted a cross-sectional survey among board-certified plastic surgeons in Saudi Arabia, collecting data through a self-administered online questionnaire. This questionnaire, which covered their demographic information and their antiseptic practices during breast implant surgery, was disseminated via a WhatsApp (Menlo Park, CA) broadcast message from May 15 to June 27, 2023.

**Results:**

Of the 52 Saudi plastic surgeons who completed the questionnaire, all reported employing preoperative antibiotics and skin disinfection. Other measures included pocket irrigation (86.5%), implant irrigation (92.3%), sleeve/funnel usage (65.4%), nipple shield usage (51.9%), and glove change during the procedure (96.2%). Nearly, all respondents used only a surgical cap for head cover (96.2%) and postoperative antibiotics as prophylaxis (98.1%). However, more than half of them did not minimize door movement during the procedure (51.9%).

**Conclusions:**

This study offers a valuable insight into the antiseptic practices during breast implant surgery in Saudi Arabia. The findings underline the need for further research to establish evidence-based guidelines for antiseptic practices in this field.

**Level of Evidence: 5:**

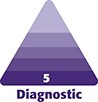

Implant-based breast reconstruction and cosmetic breast surgery are among the most frequently performed procedures by plastic surgeons worldwide. In the United States, breast augmentation is one of the most common cosmetic surgical procedures, and implant-based reconstruction is the predominant method of breast reconstruction.^1^ However, infection is a prevalent complication of implant-based breast reconstruction.^2^ Breast implant infections can lead to increased rates of capsular contracture, unhealed wounds, suboptimal aesthetic outcomes, and reconstruction failure.^3-7^

Several antiseptic measures, including prophylactic antibiotics, pocket irrigation/implant irrigation, preoperative skin disinfection, and funnel implant insertion, have been shown to reduce the risk of infection and its complications.^8-12^ However, not all plastic surgeons choose to use all these antiseptic measures, which could potentially impact the risk of implant contamination in patients. Bletsis and Der Lei investigated the trends of antiseptic measures in the Netherlands. They found that Dutch plastic surgeons employ a multitude of measures intended to reduce bacterial contamination during breast implant procedures.^13^ However, the majority of these measures remain contentious due to the limited evidence in the literature. Inspired by their work, we conducted this study to examine the trends among plastic surgeons in Saudi Arabia.

This study aims to identify the types of antiseptics commonly used among Saudi plastic surgeons. Despite the significance of this topic and its potential to prevent breast implant infections, there is a dearth of literature addressing this question. This study will provide valuable insights into the antiseptic measures used and their potential impact on patient outcomes.

## METHODS

### Ethical Considerations and Study Conduct

This study was conducted under the ethical approval and guidance of the Institutional Review Board and Research Ethics Committee. Prior to participation, all participants provided informed consent in accordance with the principles of the Helsinki Declaration. The conduct of this study was guided by the STROBE checklist.^14^

### Study Design and Sampling

In this study, we utilized a cross-sectional survey to evaluate the antiseptic measures used in breast implant surgery by Saudi plastic surgeons. The study criteria excluded residents and nonboard-certified surgeons, focusing solely on Saudi plastic surgery consultants. All participants were assured of their confidentiality and willingly participated in the study. Participation was contingent upon signing an informed consent form.

### Survey Tool and Data Collection

Data were collected between May 15 and June 27, 2023, using a self-administered survey questionnaire that was shared over WhatsApp (Menlo Park, CA). The questionnaire, structured in English, was created using Google Forms. The interface clearly outlined the purpose of the study. The authors developed the questionnaire for this study, drawing inspiration from a previously published study that shared similar aims and objectives.^13^ While the original questionnaire was not validated by the authors of the previous study, we decided to enhance the robustness of our study by validating the questionnaire. This validation process ensured that the questions were clear, relevant, and accurately measured the antiseptic practices of the surgeons. The questionnaire was divided into 2 sections. The first section included 7 demographic questions, such as gender, years of experience, type of institution, residency training program, fellowships, region of practice, type of procedure, and the number of procedures performed per year. The second section consisted of 14 questions, the majority of which focused on the antiseptic precautions used before or during breast implant surgery.

### Questionnaire Validation and Reliability

The questionnaire underwent a rigorous validation process to ensure its relevance and accuracy. A panel of 3 experts reviewed the questionnaire items in relation to the study objectives to assess their content validity for all questionnaire domains, primarily focusing on the knowledge domain. Initially, the assessment was conducted independently, and items that sparked debate were discussed in detail until consensus was reached. All suggested changes were implemented to enhance the questionnaire's validity, resulting in the final format used in the current study. The reliability of the questionnaire items was also evaluated. The questionnaire demonstrated a satisfactory level of internal consistency, with a Cronbach’s Alpha coefficient for scale data of 0.73.^15,16^ Removing any of the questionnaire items would not improve its reliability, as all postremoval Cronbach's Alpha values were below the overall estimated level of 0.74. Therefore, all items were retained. The questionnaire also exhibited a high level of stability, as evidenced by the intraclass correlation coefficient which ranged from 0.577 to 0.814.^17^ This indicates high stability and clarity of the questionnaire items (Table 1).

**Table 1.** Internal Consistency, Test–Retest Reliability, and Validation of Study Questions

Item–total statistic items	Internal consistency			Test–retest reliability
	Scale variance if item deleted	Corrected item–total correlation	Cornbrash's alpha if item deleted	ICC
Preoperative antibiotics	2.9	0.15	0.11	0.741
Pocket irrigation	3.9	0.17	0.18	0.658
If Yes to the previous question, which type of pocket irrigation?	4.6	0.09	0.29	0.747
Implant irrigation	3.4	0.47	0.27	0.804
If Yes to the previous question, which type of implant irrigation?	3.7	0.12	0.11	0.667
Skin disinfection	4.8	0.17	0.26	0.578
If yes to the previous question, which type of skin disinfection?	3.6	0.11	−0.11	0.771
Sleeve/funnel	4.1	0.17	0.08	0.577
If yes to the previous question did you use	5.0	0.24	0.26	0.716
Nipple shield	5.2	0.11	0.30	0.814
Glove change	5.0	0.24	0.26	0.702
if Yes to the previous question did you use	3.6	0.11	0.17	0.669
Door movement minimization	5.0	0.24	−0.26	0.717
Head cover	3.1	0.11	0.17	0.806

ICC, intraclass correlation coefficient.

### Statistical Analysis

The data were processed and analyzed using IBM’s Statistical Packages for Social Sciences (SPSS) version 24 (Armonk, NY). The entire respondent group was characterized using descriptive statistics, which included counts, proportions (percentages), mean, and standard deviation. A multiple response analysis was conducted to scrutinize participants’ responses to antiseptic techniques. Descriptive statistics were employed to evaluate the frequencies and percentages of participants’ responses. A *P*-value of .05 with a 95% confidence interval was used as the threshold for statistical significance.

## RESULTS

### Sociodemographic Characteristics

We received a total of 52 responses for our survey, yielding a response rate of 69.33%. The majority of our respondents were male (90.4%), falling within the age ranges of 45 to 60 years old and 30 to 45 years old (42.3% and 40.4%, respectively). Almost half of the respondents were graduates of Saudi residency programs (46.2%), with a significant portion having completed aesthetic fellowships (42.3%). Most respondents had 6 to 10 years of postresidency or fellowship training (25.0%), with over half practicing in the central region (51.9%). The majority were employed in nonacademic hospitals (36.5%) and performed both breast augmentation and reconstruction procedures (76.9%). The frequency of these procedures was either 10 to 50 per year or <10 per year (61.5% and 50.0%, respectively). Further details about the sociodemographic characteristics of the respondents can be found in Table 2.

**Table 2.** Demographic Information of Included Plastic Surgeons

Parameter	Category	Overall, *n* = 52
1. Age	30 to <45	21 (40.4%)
	45 to <60	22 (42.3%)
	60 or more	9 (17.3%)
2. Gender	Male	47 (90.4%)
	Female	5 (9.6%)
3. Residency training program	Saudi	24 (46.2%)
	French	7 (13.5%)
	Canadian	15 (28.8%)
	German	1 (1.9%)
	Other	5 (9.6%)
4. Fellowships	Aesthetic	22 (42.3%)
	Burn	3 (5.8%)
	Craniofacial	2 (3.8%)
	Hand Surgery	9 (17.3%)
	Microsurgery	5 (9.6%)
	Pediatric plastic surgery	2 (3.8%)
	Breast	9 (17.3%)
5. How many years have you been in practice postresidency or fellowship training?	0 to 5 years	11 (21.2%)
	6 to 10 years	13 (25.0%)
	11 to 15 years	10 (19.2%)
	16 to 20 years	5 (9.6%)
	21 to 25 years	5 (9.6%)
	>25 years	8 (15.4%)
6. Region of practice	Central	27 (51.9%)
	Western	19 (36.5%)
	Eastern	6 (11.5%)
7. Primary practice setting	Private practice	16 (30.8%)
	Academic	3 (5.8%)
	Nonacademic hospital	19 (36.5%)
	Private practice and academic	7 (13.5%)
	Private practice and nonacademic hospital	6 (11.5%)
	Private practice and academic and nonacademic hospital	1 (1.9%)
8. Procedure	Breast augmentation	12 (23.1%)
	Breast reconstruction	0 (0%)
	Both	40 (76.9%)
9. Breast augmentation cases (per year)	<10	9 (17.3%)
	10-50	32 (61.5%)
	>50	11 (21.2%)
10. Breast reconstruction cases (per year)	<10	26 (50.0%)
	10-50	25 (48.1%)
	>50	1 (1.9%)

### Antiseptic Measures Usage

In terms of antiseptic measures used by Saudi plastic surgeons, all respondents (100%) reported the use of preoperative antibiotics. Pocket irrigation was used by 86.5% of respondents, with 42.3% using both povidone-iodine (PI; Purdue Pharma L.P., Stamford, CT) and antibiotics. Implant irrigation was used by 92.3% of respondents, with 38.5% using antibiotics only. Skin disinfection was used by all respondents (100%), with PI being the most common disinfectant (80.8%).

Other measures included the use of a sleeve/funnel (65.4%), with 25.0% using antibiotics only, and a nipple shield (51.9%). Glove change was common practice (96.2%), while over half of the respondents did not minimize door movement (51.9%). Only surgical caps were used as head covers (96.2%), while (3.8%) of our surgeons used both surgical cap and beard cover. Most respondents (98.1%) used antibiotics postoperatively as a prophylactic course. More details about antiseptic usage are provided in Table 3.

**Table 3.** Antiseptic Measures and Irrigation Substances

Parameter	Category	Overall, *n* = 52
1. Preoperative antibiotics	Yes	52 (100.0%)
	No	0 (0%)
2. Pocket irrigation	Yes	45 (86.5%)
	No	7 (13.5%)
3. If Yes to the previous question, which type of pocket irrigation?	Povidone-iodine	8 (15.4%)
	Antibiotic	11 (21.2%)
	Both	22 (42.3%)
	Other	5 (9.6%)
4. Implant irrigation	Yes	48 (92.3%)
	No	4 (7.7%)
5. If Yes to the previous question, which type of implant irrigation?	Povidone-iodine	10 (19.2%)
	Antibiotic	20 (38.5%)
	Both	16 (30.8%)
	Other	3 (5.8%)
	No response	3 (5.8%)
6. Skin disinfection	Yes	52 (100.0%)
	No	0 (0%)
7. If Yes to the previous question, which type of skin disinfection?	Povidone-iodine	42 (80.8%)
	Chlorhexidine	3 (5.8%)
	Povidone-iodine and chlorhexidine	5 (9.6%)
	No response	2 (3.8%)
8. Sleeve/funnel	Yes	34 (65.4%)
	No	18 (34.6%)
9. If yes to the previous question, did you use:	Povidone-iodine	7 (13.5%)
	Antibiotic	13 (25.0%)
	Both	11 (21.2%)
	Other	3 (5.8%)
	No response	18 (34.6%)
10. Nipple shield	Yes	27 (51.9%)
	No	25 (48.1%)
11. Glove change	Yes	50 (96.2%)
	No	2 (3.8%)
12. Door movement minimization	Yes	25 (48.1%)
	No	27 (51.9%)
13. Head cover	Surgical cap	50 (96.2%)
	Surgical hood + beard cover	2 (3.8%)
14. Antibiotics postoperatively as a prophylactic course?	Yes	51 (98.1%)
	No	1 (1.9%)

When considering the most commonly used antiseptic measures among Saudi plastic surgeons, preoperative antibiotics and skin disinfection were the most prevalent. Conversely, the use of a nipple shield and minimization of door movement were the least common practices (Figure).

**Figure.**
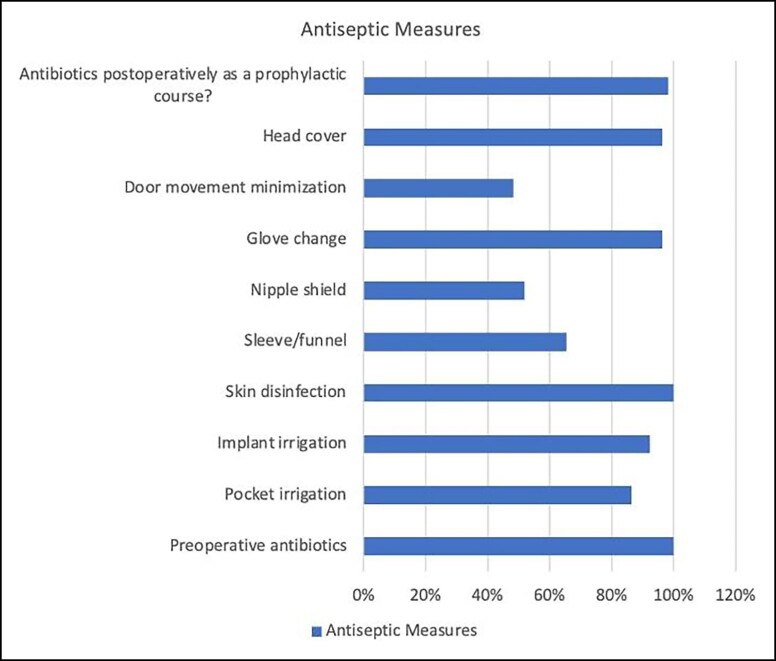
The proportions of antiseptic measures used by plastic surgeons.

### Association Between Demographic Characteristics and Antiseptic Measures

There was a significant correlation between age and the use of head coverings (*P* = .014). The type of residency training program also showed a significant correlation with the use of implant irrigation and head coverings (*P* = .002 for both). The number of years postresidency was significantly associated with the use of a sleeve/funnel and head coverings (*P* = .007 and *P* = .043, respectively). Practicing in the central region was significantly associated with the use of a sleeve/funnel, minimization of door movement, and the use of postoperative antibiotics as a prophylactic course (*P* = .007, *P* = .001, and *P* = .040, respectively). More details are summarized in Table 4.

**Table 4.** Association Between Demographic Characteristics and Antiseptic Measures

*N*= 52		Pocket irrigation	Implant irrigation	Sleeve/funnel	Nipple shield	Glove change	Door movement minimization	Head cover	Antibiotics postoperative as prophylactic course?
Age	Pearson correlation	−0.186	0.092	0.176	0.20	0.064	−0.20	0.340^a^	0.045
	Significance	0.188	0.516	0.212	0.15	0.653	0.154	0.014	0.753
Gender	Pearson correlation	−0.129	−.094	0.174	0.078	−0.065	0.053	−0.065	−0.046
	Significance	0.363	0.507	0.217	0.583	0.646	0.710	0.646	0.748
Residency training program	Pearson correlation	0.041	0.417^b^	0.082	0.007	0.095	−0.256	0.418^b^	0.066
	Significance	0.775	0.002	0.561	0.962	0.505	0.067	0.002	0.641
Fellowships	Pearson correlation	−0.070	−0.190	−0.050	0.088	0.150	0.345^a^	−0.067	0.226
	Significance	0.621	0.178	0.726	0.537	0.290	0.012	0.639	0.107
How many years have you been in practice postresidency or fellowships training	Pearson correlation	−0.149	0.239	0.367^b^	0.158	0.107	−0.158	0.282^a^	−0.006
	Significance	0.293	0.088	0.007	0.262	0.449	0.262	0.043	0.965
Region of practice	Pearson correlation	−0.096	0.065	0.369^b^	0.061	−0.174	−0.454^b^	0.118	0.286^a^
	Significance	0.497	0.649	0.007	0.665	0.218	0.001	0.406	0.040
Primary practice setting	Pearson correlation	−0.010	−0.102	0.329^a^	−0.238	−0.248	−0.061	−0.177	0.025
	Significance	0.944	0.471	0.017	0.089	0.077	0.666	0.209	0.862

a Correlation is significant at the .05 level (2-tailed). ^b^Correlation is significant at the .01 level (2-tailed).

## DISCUSSION

Breast implant surgery is one of the most common procedures performed by plastic surgeons in the United States,^1^ with capsular contracture and infections being frequent complications.^3-7^ The usage of antiseptic measures varies widely among surgeons, as there is no consensus on the matter. In our study, we found that all surgeons (100%) used perioperative antibiotics and skin disinfection. Proper skin disinfection has been shown to decrease breast contamination and even capsular contracture.^11^ Although there are no specific guidelines for perioperative prophylaxis in breast implant surgery, a Cochrane review indicates that preoperative antibiotics significantly reduce surgical-site infection in patients undergoing breast cancer surgery without reconstruction.^18^ Pocket irrigation was used by 86.5% of our surgeons, with both PI and antibiotics being used by 42.3%. Implant irrigation was used by 92.3% of surgeons. The use of pocket and implant irrigation with antibiotics and PI has been clinically proven to decrease infection and capsular contracture.^9,10^ Interestingly, our study revealed that over half of our surgeons (51.9%) did not adhere to door movement minimization precautions. There is substantial evidence suggesting that frequent door openings during surgery cause pressure imbalance, which in turn increases the risk of surgical-site infection.^19^ Head coverings were primarily used by our surgeons, with 96.2% opting for a surgical cap without a beard cover. Despite our sample consisting of 90.4% male plastic surgeons, only 3.8% used a surgical cap and beard cover. Bearded men are known to shed more bacteria compared to those who are clean shaven.^20^ Glove changing during surgery was adhered to by 96.2% of surgeons, which is crucial as wearing gloves for longer than 90 min increases the risk of perforation.^21^ The use of a sleeve/funnel was reported by 65.4% of our surgeons, a figure that contradicts a previous study showing that only 17.2% of Dutch plastic surgeons use a sleeve/funnel.^13^ This variation may be due to the lack of sufficient evidence supporting the use of a funnel/sleeve to decrease breast implant contamination. Many antiseptic measures are used based on clinical judgment, despite the lack of strong supporting evidence. This study provides valuable insight into the adherence of Saudi plastic surgeons to antiseptic measures. There is a need for further research to validate the use of different types of antiseptics, with the ultimate goal of formulating guidelines to standardize the use of antiseptic measures in breast implant surgery.

### Limitations and Future Directions

There are certain limitations in this study that should be acknowledged. Firstly, the reliance on self-reported survey data introduces the possibility of bias, as respondents may be subject to recall and interpretation biases. Secondly, nonresponse bias may have influenced our findings. However, considering the relatively high response rate (69.33%), we believe that our results provide an accurate reflection of the current practices of plastic surgeons in Saudi Arabia. Lastly, this study primarily examined the use of antiseptic measures but did not evaluate their impact on patient outcomes, such as infection rates or complication rates. While the study provides valuable insights into the prevalence of antiseptic measure usage among Saudi plastic surgeons, further research is needed to establish the association between these measures and clinical outcomes. In addition to the limitations mentioned above, we recommend that future studies assess whether there is an association between a plastic surgeon’s number of complications or infection rates and their specific antiseptic techniques. This would provide valuable insights into the effectiveness of different antiseptic measures in preventing complications and infections in breast implant surgery. By examining the relationship between antiseptic techniques and patient outcomes, further research can contribute to the development of evidence-based guidelines and recommendations for antiseptic measure usage in plastic surgery.

## CONCLUSIONS

This study reveals the diverse techniques employed by Saudi plastic surgeons in utilizing antiseptic measures during breast implant surgery. However, it is evident that many of these measures lack substantial evidence. The development of comprehensive guidelines is crucial to establish a standardized approach to antiseptic measure usage among plastic surgeons. Future research should concentrate on evaluating the impact of these measures on patient outcomes and exploring innovative approaches. By addressing these gaps, we can enhance patient safety and foster the adoption of evidence-based practices in the field of plastic surgery.
